# Quantification of autoantibodies using a luminescent profiling method in autoimmune interstitial lung disease

**DOI:** 10.3389/fimmu.2024.1462242

**Published:** 2024-10-25

**Authors:** Peter D. Burbelo, Julio A. Huapaya, Zohreh Khavandgar, Margaret Beach, Iago Pinal-Fernandez, Andrew L. Mammen, John A. Chiorini, Payam Noroozi Farhadi, Frederick W. Miller, Adam Schiffenbauer, Kakali Sarkar, Blake M. Warner, Lisa G. Rider

**Affiliations:** ^1^ Adeno-Associated Virus Biology Section, National Institute of Dental and Craniofacial Research, National Institutes of Health, Bethesda, MD, United States; ^2^ Critical Care Medicine and Pulmonary Branch, National Heart, Lung and Blood Institute, National Institutes of Health, Bethesda, MD, United States; ^3^ Sjögren’s Disease Clinic, National Institute of Dental and Craniofacial Research, National Institutes of Health, Bethesda, MD, United States; ^4^ Muscle Disease Section, National Institute of Arthritis and Musculoskeletal and Skin Diseases, National Institutes of Health, Bethesda, MD, United States; ^5^ Department of Neurology, Johns Hopkins University School of Medicine, Baltimore, MD, United States; ^6^ Environmental Autoimmunity Group, National Institute of Environmental Health Sciences, National Institutes of Health, Bethesda, MD, United States; ^7^ Salivary Disorders Unit, National Institute of Dental and Craniofacial Research, National Institutes of Health, Bethesda, MD, United States

**Keywords:** interstitial lung disease, myositis-specific autoantibody, myositis-associated autoantibody, idiopathic inflammatory myopathies, Sjögren’s disease

## Abstract

Autoantibodies are important for the diagnosis of autoimmune interstitial lung disease (ILD). Standard immunoassays have limitations, including their qualitative nature and/or a narrow dynamic range of detection, hindering the usefulness of autoantibodies as biomarkers of disease activity. Here, the luciferase immunoprecipitation system (LIPS) was evaluated for measuring myositis-specific and other lung-related autoantibodies in 25 subjects with idiopathic inflammatory myopathies (IIM), 26 with Sjögren’s disease (SjD), and 10 healthy volunteers. LIPS detected a broad dynamic range of autoantibodies, to MDA5, Jo-1, PL12, KS, U1-70K, and Ro52, and matched seropositivity status with established immunoassays. Robust anti-MDA5 autoantibodies in four IIM-ILD patients had a median value of 1,134,000 LU (IQR 473,000-2,317,000), which was 500 times higher than in 21 seronegative IIM patients. Markedly elevated anti-Jo-1 autoantibodies in five IIM-ILD patients demonstrated a median value of 1,177,000 LU (IQR: 604,000-2,520,000), which was 1000-fold higher than in seronegative patients. Robust anti-Ro52 and other anti-tRNA-synthetase autoantibodies were detected in a subset of IIM-ILD subjects. In SjD, only anti-U1-70K and KS autoantibodies were identified in ILD patients with a prevalence of 30% and 20%, respectively. In longitudinal samples of five IIM-ILD patients, anti-Jo-1 autoantibody levels paralleled clinical improvement of lung function. LIPS can accurately quantify autoantibody levels as biomarkers for treatment response in patients with autoimmune ILD.

## Introduction

Interstitial lung disease (ILD) represents a diverse group of disorders sharing radiographic, physiologic, and histopathologic pulmonary manifestations (1). In systemic autoimmune diseases such as idiopathic inflammatory myopathies (IIM) and Sjögren’s disease (SjD), ILD is a frequent manifestation associated with high morbidity and mortality (2). Autoantibodies play an important role in the diagnosis of these diseases and have contributed to more specific clinical characterization of these patients (3–6). For example, patients with anti-PL7 and anti-PL12 autoantibodies present with more severe lung involvement than those with anti-Jo1 (6). Anti-MDA5 autoantibodies confer an increased risk of rapidly progressive ILD (5). A high prevalence of sicca symptoms has been reported in patients with anti-KS autoantibodies (7).

Recent evidence also suggests autoantibodies are not only useful biomarkers (8, 9), but they play an important role in the disease pathogenesis of IIM (10, 11) and IIM-ILD (8, 12). For example, treatment with CAR-T CD19 cells can reduce autoantibody titers and induce sustained remission in patients with anti-synthetase syndrome (13). Additionally, IIM patients who respond to rituximab therapy also experience a reduction in autoantibody levels (14). Importantly, research quantifying autoantibody levels as relevant disease biomarkers has been limited by several factors. While immunoprecipitation (IP) is considered the gold standard test, this immunoassay is costly, slow to generate results, unable to differentiate between autoantibodies against autoantigens with similar molecular weights, and does not provide autoantibody levels. Standard assays, including ELISA, Western blots, and line blot immunoassays (LIA) also have limitations in accurately determining autoantibody-positive and -negative patients, and/or the accurate quantification of autoantibody levels (15). For instance, a recent study by Loganathan et al. highlighted the failure of standard assays to detect non-Jo-1 antisynthetase autoantibodies, such as PL7, OJ, and KS, with high sensitivity and specificity compared to IP (16).

Our group developed Luciferase immunoprecipitation systems (LIPS) as a simple, quantitative immunoassay that employs light-emitting proteins in an immunoprecipitation format for the detection of autoantibodies against both linear and conformational epitopes (17). We have shown in multiple studies that LIPS has high sensitivity and specificity for detecting and quantifying autoantibodies associated with a multitude of autoimmune conditions, including systemic sclerosis (18), systemic lupus erythematosus (SLE) (19), and SjD (17, 20). In particular, LIPS has shown to provide a wide range of anti-Ro52 autoantibody levels (21, 22), which is a myositis-associated autoantibody that has been associated with IIM-ILD (23–27). In this study, LIPS was used to detect and quantify myositis-specific and myositis-associated autoantibodies in patients with IIM and SjD.

## Materials and methods

### Study design and patient characteristics

Sera were collected at the National Institutes of Health Clinical Research Center from patients enrolled in institutional review board-approved protocols (NIEHS: 94-E-0165, 11-E-0072, 07-E-0012, 05-E-N200; NIDCR: 15-D-0051, 11-D-0172) after informed consent was obtained. Since the focus of this study was to identify biomarkers of ILD, the IIM group was pre-selected to include a large percentage of patients with known ILD. The study included cross-sectional serum samples from 10 healthy volunteers (NV), 25 patients with IIM (21 with ILD), and 26 SjD patients (10 with ILD). The IIM patient group (n=25) were enrolled based on the criteria of Bohan and Peter (28) for myositis, in which 14 met definite EULAR-ACR criteria (29). Among the IIM-ILD subjects, 42% (9/21) had identifiable ILD subtypes based on lung biopsy and/or high-resolution computed tomography (HRCT) reports. Specifically, six subjects had nonspecific interstitial pneumonia (NSIP), two subjects had cryptogenic organizing pneumonia/bronchiolitis obliterans organizing pneumonia (COP/BOOP), and one subject had both NSIP and Organizing Pneumonia (OP) on lung biopsy. All the SjD participants used in this study met the 2016 American College of Rheumatology Sjögren’s Disease Classification Criteria including assessment of focal lymphocytic (range 0-12). Among the ten SjD-ILD cases, 70% (7/10) had identifiable ILD subtypes. Three SjD-ILD subjects had non-specific fibrosis, two had NSIP, one with acute interstitial pneumonitis and one with lymphocytic interstitial pneumonitis. All participants in this study were comprehensively evaluated, including for ILD, and had clinical autoantibody testing. IIM patients also had a physician global activity (PGA) score, on a 10 cm visual analog scale (30).

In addition to cross-sectional patient samples, anti-Ro52, and anti-Jo-1 autoantibody levels were analyzed in 5 IIM patients with longitudinal samples. Clinical and lung function measurements, including forced vital capacity, percent predicted (FVC %) and diffusing capacity of the lungs for carbon monoxide percent predicted (DLCO), for all available serial samples for these patients were also included in analysis.

### LIPS measurement of autoantibody levels in the cohort

The LIPS immunoassay was used in a 96-well format to detect autoantibodies to both conformational and linear epitopes of protein antigens (17). The technology involves the use of custom luciferase-antigen fusion proteins employed in a fluid-phase immunoassay which provides high sensitivity, specificity, and a wide dynamic range of detection. Several previously described luciferase-fusion proteins for LIPS were used against known autoantigens including Ro52, Ro60, CENP-A, U1-70K, Jo-1, IFN-α, IFN-ω, KCNRG, BPIFB1 and TRIM38 (19, 31, 32). For this study, new autoantigen fusion proteins were constructed for detecting autoantibodies against MDA5 (IFIH-1), PL7 (TARS1), PL12 (AARS1), EJ (GARS1), KS (NARS1), Ha (YARS1), OJ (IARS1), Zo (FARS1), ABLIM (actin-binding LIM protein 1) and CDH5R (cadherin-5). cDNAs were amplified from either existing plasmid clones or generated as synthetic DNA fragments (Twist Bioscience) and were then cloned as C-terminal fusion proteins with *Renilla* luciferase except for CDH5R, which were generated as N-terminal fusion with *Gaussia* luciferase. The exact amino acids (aa) used for the new target autoantigens are as follows: MDA5/IFIH1 (NP_071451.2) with a N-terminal protein fragment spanning aa 2-577 and a C-terminal fragment spanning aa 578-1105), PL7/TARS1 (NP*_*001245366.1); aa 281-723, PL12/AARS1 (NP_001596.2); aa 516-968, EJ/GARS1 (NP_002038.2); aa 149-739, KS/NARS1 (NP_004530.1); aa 1-548, Ha/YARS1 (NP_003671.1); aa 2-528, OJ/IARS1 (NP_001365515.1); aa 679-1211, Zo/FARS-beta subunit (NP_005678.3); aa 2-598, ABLIM (NP_001309817.1.2); aa 2-455, and CDHR5 (NP_001165439.2); aa 1-520. Bacterial cultures containing these plasmids were expanded and plasmid DNA was prepared using a Qiagen Midi kit. The purified plasmids were then used for DNA sequencing to confirm construct integrity and for transfection of mammalian cells.

As previously described (33), plasmids for the mammalian expression vectors encoding different luciferase autoantigen fusion protein constructs were transfected into Cos1 cells with Lipofectamine 2000, and cell lysates were harvested 48 hours later to obtain crude cell extracts. For autoantibody testing, serum samples were diluted 1:10 in assay buffer A (20 mM Tris, pH 7.5, 150 mM NaCl, 5 mM MgCl_2_, 1% Triton X-100) and diluted aliquots (10 μl) were then evaluated using a 96-well plate format. For these tests, serum (equivalent to 1 μl of serum), 40 μl of buffer A and 50 μl of Cos1 cell extract containing 10^7^ light units (LU) of a particular luciferase*-*antigen extract were used. After incubation at room temperature for one-hour, a microtiter filter plate containing protein A/G beads captured the IgG antibody-antigen complexes during a one-hour incubation. The antibody-antigen-bead complexes were then washed eight times with buffer A and twice with PBS on a microtiter filter plate to remove unbound antigens. After the final wash, LU were measured in a Berthold LB 960 Centro microplate luminometer (Berthold Technologies, Bad Wildbad). Coelenterazine substrate (Promega) was used for the detection of *Renilla* luciferase and *Gaussia* luciferase reporter activity and the Nano-Glo^®^ substrate (Promega) was used for nanoluciferase (33).

### Comparison of LIPS with other immunoassays

The serological results for the LIPS assay for the anti-MDA5 and anti-aminoacyl-tRNA-synthetase autoantibodies in the IIM group were compared with testing at the University of Pittsburgh or OMRF by immunoprecipitation and immunoprecipitation-immunoblot (34). The Ro52 LIPS results were compared with anti-Ro52 autoantibody testing by ELISA as described (23). Except for KS testing, all samples were evaluated by LIPS without knowledge of this serological immunoreactivity information. *Post-hoc* comparison with clinical testing was compared where applicable.

### Statistical analysis

GraphPad Prism software (San Diego, CA) was used for analyzing the autoantibody levels in this study. Autoantibody levels, expressed as median log (10) LU and 25-75% interquartile range (IQR), were calculated, and presented as antilog values. The non-parametric Mann-Whitney *U* statistical test was used for comparison of autoantibody levels in the different subject groups. Calculations of sensitivity and specificity for the different LIPS assays involved cut-off limits derived from previous studies (18) or from the mean LU value plus five standard deviations based on values obtained from the normal controls.

A colored heatmap was used to compare the relative autoantibody levels between the different patients for each of the different seropositive antigens. The mean plus five standard deviations cutoff value based on the healthy volunteers was first subtracted from the autoantibody levels for each antibody-serum pair. The resulting value was then divided by the corresponding standard deviation for the specific autoantibody to yield a relative level of the autoantibody above these baseline values and was then color-coded from pink to dark black.

## Results

### Characteristics of the subjects in the cohort with and without ILD

The cohort of subjects studied included 26 with SjD, 25 with IIM, along with 10 healthy volunteers as a reference group (**Table 1**). Ten (38%) of the SjD and 21 (84%) of the IIM patients had ILD, respectively. Among the IIM group, 15 patients were classified with adult dermatomyositis (ADM) (one also had overlapping autoimmune thyroid disease), 6 with adult polymyositis (APM), 3 participants with juvenile dermatomyositis (JDM) (one with overlapping scleroderma), and one with juvenile polymyositis (JPM) overlapping with SLE. Within the SjD group, determination of the focus score, a marker of inflammation within the salivary gland, showed that the mean focus score in the SjD without and with ILD was 4.8 and 3.0, respectively.

**Table 1:** Demographic and clinical information for the study groups.

	SjD (n=26)	IIM (n=25)	NV (n=10)
Sex (% Female)	92% (24)	80% (20)	60% (6)
Age (years) ± SD	52.8± 14.0	39 ± 20.0	44.4 ± 13.1
Race			
White	17	16	5
Black	3	6	4
Other	6	3	1
Clinical Diagnosis			
Adult dermatomyositis	-	15	-
Adult polymyositis	-	6	-
Juvenile dermatomyositis	-	3^b^	-
Juvenile polymyositis		1^b^	
Interstitial Lung Disease (n)	10	21	-
Treatment			
Steroids	14	21	-
Immunosuppression	12	22	-

^a^One patient had autoimmune thyroid disease.

^b^One with juvenile dermatomyositis had overlapping scleroderma, and one with juvenile polymyositis had overlapping systemic lupus erythematosus.

SjD, Sjögren’s syndrome; IIM, idiopathic inflammatory myopathy; NV, normal volunteer; SD, standard deviation.

### Detection of aminoacyl-tRNA synthetase autoantibodies by LIPS

**Figure 1:**
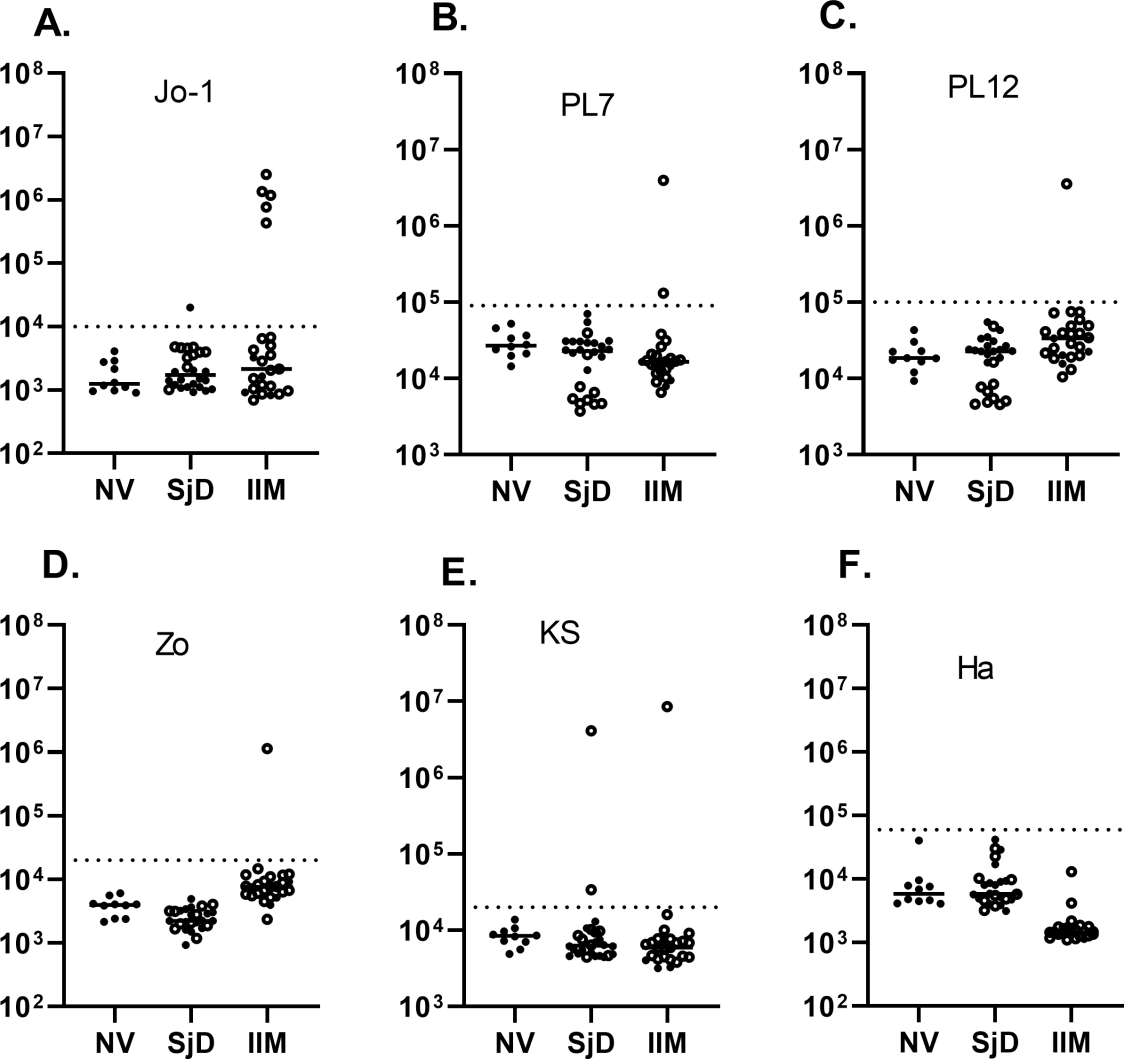
Autoantibodies against a panel of aminoacyl-tRNA synthetases. Autoantibody levels by LIPS were determined against six aminoacyl-tRNA synthetases including **(A)** Jo-1, **(B)** PL7, **(C)** PL12, **(D)** Zo, **(E)** KS, and **(F)** Ha. Each symbol represents a sample from subjects who were healthy volunteers (NV) or diagnosed with SjD (Sjogren’s disease) and IIM (idiopathic inflammatory myopathy). SjD and IIM subjects with interstitial lung disease are shown by the open circles. Autoantibody levels are plotted in light units on a log_10_ scale, and the dashed lines represent the cut-off level for determining seropositive autoantibodies for each antigen, as described in the Methods.

Autoantibodies against eight different aminoacyl-tRNA-synthetases were tested in the cohort. Among the anti-aminoacyl-tRNA-synthetase autoantibodies examined, anti-Jo-1 autoantibody was the most prevalent in our IIM cohort, with 5 patients (20%) displaying seropositivity. The median level for anti-Jo-1 autoantibodies was 1,176,600 LU and included a wide dynamic range (IQR: 604,000-1,940,000). In contrast, seronegative IIM patients exhibited a significantly lower median anti-Jo-1 autoantibody level of 1,586 LU (IQR: 930-3490) (**Figure 1A**). Analysis of anti-PL7 autoantibodies identified two seropositive IIM patients (**Figure 1B**). Notably, one patient exhibited high autoantibody levels, while another displayed levels near the cut-off value. The assessment of other tRNA-synthetase autoantibodies revealed one patient with anti-PL12 autoantibodies (**Figure 1C**), one with anti-Zo autoantibodies (**Figure 1D**), and one patient with anti-KS autoantibodies (**Figure 1E**). Of note, none of the patients within the IIM cohort exhibited anti-Ha (**Figure 1F**), anti-EJ, or anti-OJ autoantibodies (*data not shown*). Although independent testing did not examine anti-Zo autoantibodies, overall, the tRNA-synthetase autoantibody profile determined by OMRF testing closely matched the results from LIPS (**Supplementary Table 1**). However, one APM-ILD subject who had both anti-Jo-1 and anti-PL7 autoantibodies in the LIPS assay, was only positive for anti-Jo-1 autoantibodies by the OMRF immunoprecipitation assay. In the SjD cohort, one patient without clinical signs of ILD was positive for anti-Jo-1 autoantibodies (**Figure 1A**), and two patients with SjD-ILD were positive for anti-KS autoantibodies (**Figure 1E**).

### Detection of anti-MDA5 autoantibodies by LIPS

**Figure 2:**
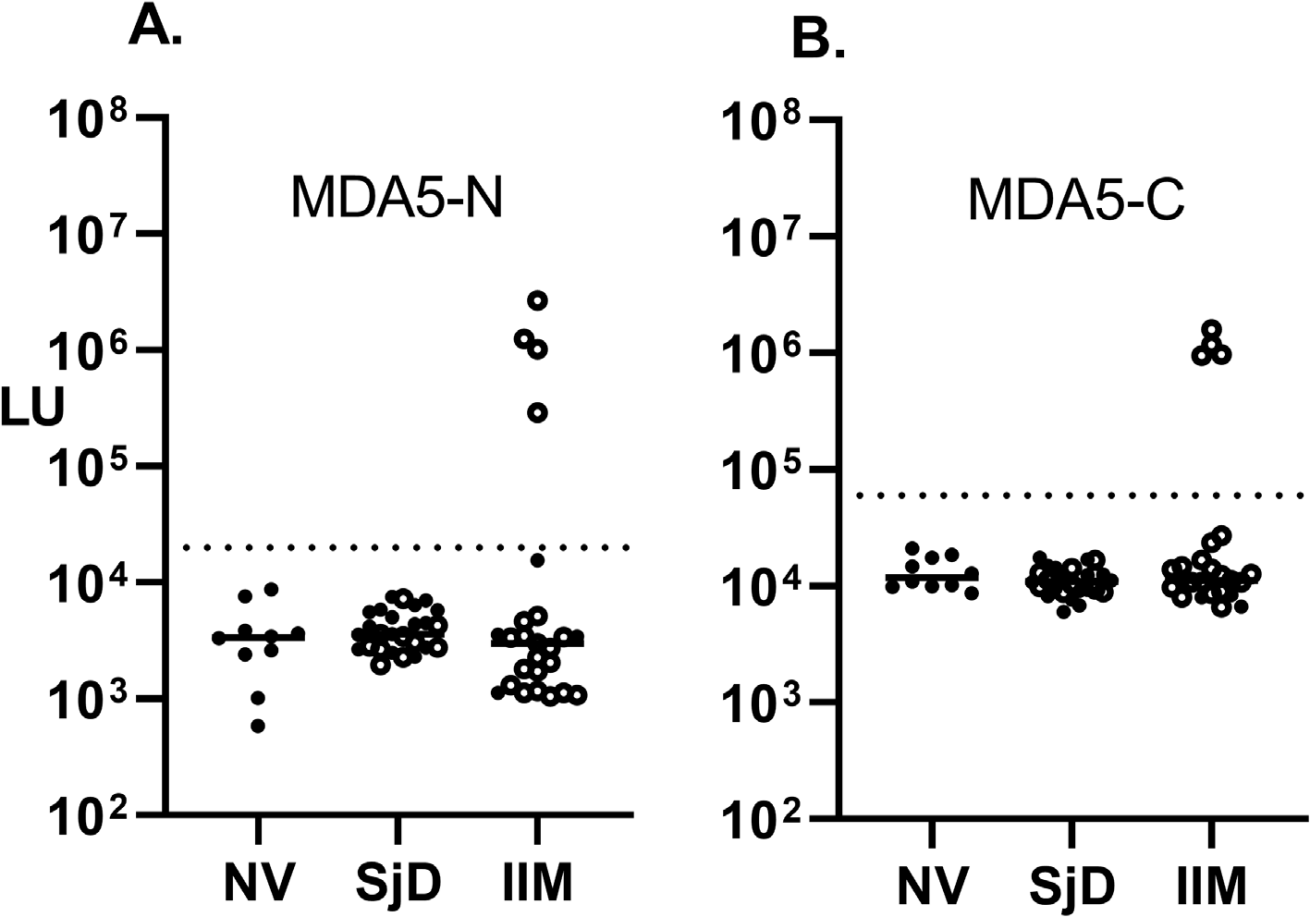
Detection of autoantibodies against the N- and C-terminal protein fragments of the MDA5 (IFIH1) autoantigen. Autoantibody levels against the **(A)** N-terminal (MDA5-N) and **(B)** C-terminal half of MDA5 protein (MDA5-C) were measured by LIPS in the cohort. Each symbol represents a sample from subjects who were healthy volunteers (NV) or diagnosed with SjD (Sjogren’s disease) and IIM (idiopathic inflammatory myopathy). Autoantibody levels are plotted in light units on a log_10_ scale, and the dashed lines represent the cut-off level for determining seropositive autoantibodies, as described in the Methods.

LIPS was also used to measure anti-MDA5 autoantibodies in the IIM and SjD cohorts. Based on the relatively large protein size of the MDA5 autoantigen, encoded by the IFIH1 gene, two protein fragments spanning the N-terminal, designated MDA5-Δ1 (aa 2-577), and C-terminal halves of the protein, designated MDA5A-Δ2 (578–1105), were generated and separately tested. Using cut-off values derived from the normal volunteers, seropositive MDA5-Δ1 autoantibodies were detected in 4 (16%) patients from the IIM group and none of SjD patients (**Figure 2A**). The median value for MDA5-Δ1 autoantibodies was 1,134,000 LU (IQR 473,000-2,316,000) in those four patients, which was approximately 100 times higher than the remaining 21 seronegative patients (median of 2,279 LU, IQR 1,147-3,472). Analysis for autoantibodies against the MDA5-Δ2 protein fragment showed similar results, identifying the same four IIM subjects as seropositive (**Figure 2B**). The comparison between the LIPS assay and the MDA5 IP-immunoblot test (OMRF) detected the same seropositive samples amongst the JDM and ADM samples (**Supplementary Table 1**).

### Autoantibodies against other targets in the IIM and SjD cohorts

**Figure 3:**
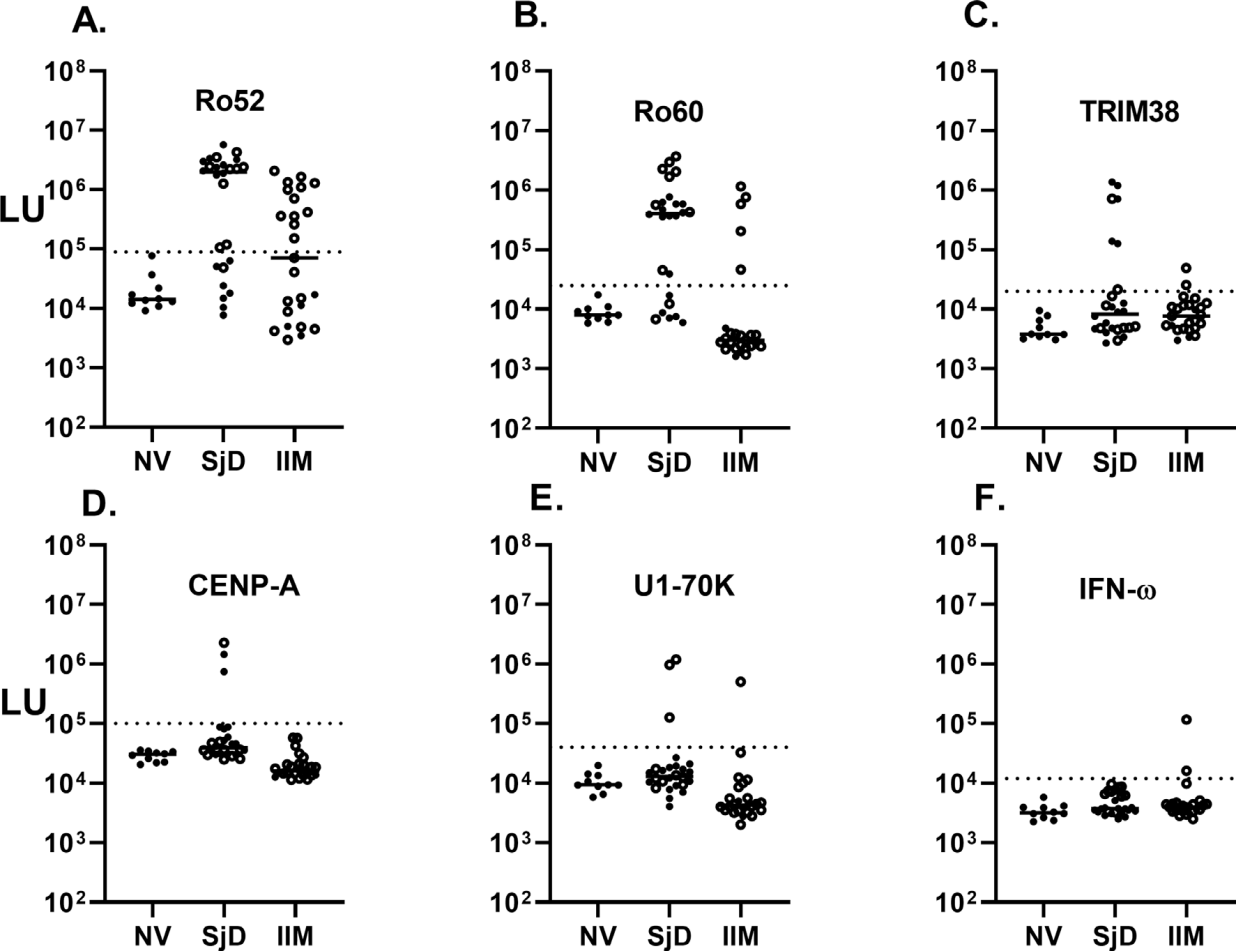
Autoantibodies against six known pulmonary-associated autoantigens by LIPS assay. Autoantibody levels against six known autoantigens including **(A)** Ro52, **(B)** Ro60, **(C)** TRIM38, **(D)** CENP-A, **(E)** U1-70K, and **(F)** IFN-omega determined by LIPS. Each symbol represents a sample from individual healthy subjects (NV) or patients with SjD and IIM. Autoantibody levels are plotted in light units on a log_10_ scale, and the dashed lines represent the cut-off level for determining seropositive autoantibodies for each antigen as described in the Methods.

In an exploratory fashion, the IIM and SjD cohorts were analyzed for autoantibodies against a panel of antigens associated with multiple autoimmune diseases, including autoimmune-associated lung disease. Anti-Ro52, a myositis-associated autoantibody, was identified in 48% (12/25) of the IIM and 61% (16/26) of the SjD subjects. These numbers increased when only patients with ILD were examined, showing as positive in 57% (12/21) of IIM-ILD and in 70% (7/10) of the SjD-ILD (**Figure 3A**). The median value for anti-Ro52 autoantibodies in the seropositive IIM and SjD subjects was 1,827,000 LU (IQR 640,000-2,500,000) compared to the remaining seronegative patients (median of 13,300 LU, IQR (4918-32,480). Ro52 autoantibody detection by LIPS exactly matched the available seropositive Ro52 status in the IIM subgroup determined by ELISA. Anti-Ro60 autoantibodies demonstrated a high prevalence in the non-ILD SjD (63%) and SjD-ILD (80%) patients but were only present in 5 (16%) patients in the IIM cohort (**Figure 3B**). TRIM38 autoantibodies, a Ro52-related molecule, were present in 30% of the non-ILD SjD cohort, 20% of the SjD-ILD cohort, and 5% of IIM-ILD patients (**Figure 3C**). Anti-CENP-A autoantibody, associated with systemic sclerosis, was only positive in two non-ILD SjD and one SJD-ILD patients (**Figure 3D**). None of the IIM patients were seropositive for anti-CENP-A autoantibody. Anti-U1-70K autoantibodies, associated with mixed connective tissue disease and overlap myositis, were seropositive in three SjD-ILD and one IIM-ILD patients (**Figure 3E**). In the IIM group, U1-70K autoantibody detection matched the OMRF immunoprecipitation seropositive status (**Supplementary Table 1**) Lastly, interferon-ω autoantibodies, associated with viral pulmonary disease, were detected in one IIM subject at relatively high levels of over 200 times higher than the controls, and at low levels in another IIM individual (**Figure 3F**). Further testing for anti-interferon-α5 autoantibodies revealed seropositivity exclusively in the one IIM individual exhibiting elevated levels of auto-IFN-ω autoantibodies, but in no other samples (*data not shown*). Autoantibody testing against two other autoantigens, KCNRG and BPIFB1, found in autoimmune polyendocrinopathy-candidiasis-ectodermal dystrophy (APECED) patients with autoimmune pneumonia, revealed no seropositivity in any of the SjD or IIM subjects (*data not shown*). Similarly, autoantibodies to two other antigens, ABLIM (35) and CDHR5 (36), associated with pulmonary disease in two recent studies identified by phage immunoprecipitation sequencing (PhIP-Seq) were seronegative by LIPS in the cohort (*data not shown*).

### Heatmap analysis of autoantibody immunoreactivity

**Figure 4:**
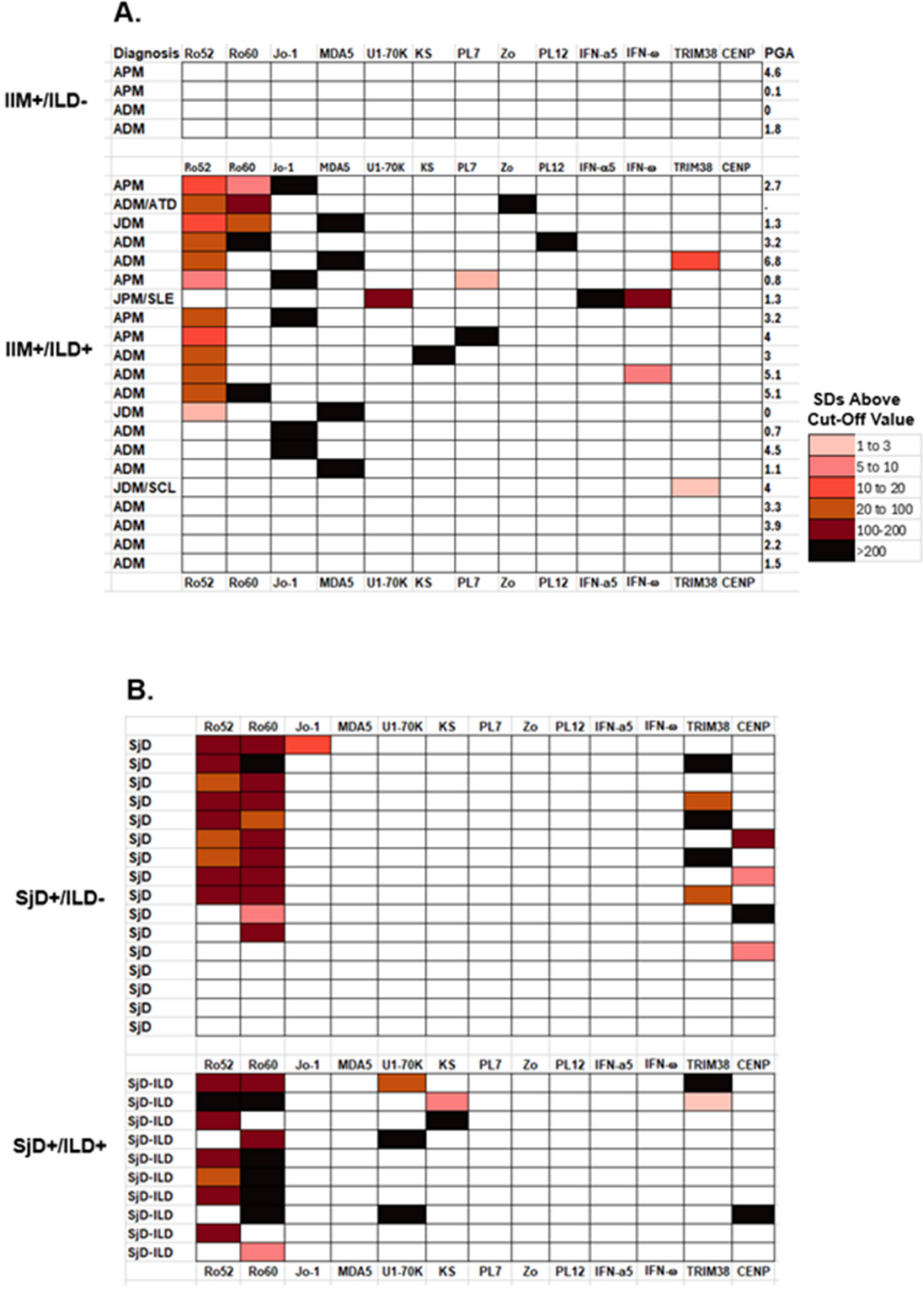
Heatmap analysis of autoantibodies in the IIM and SjD cases. Heatmap analysis shows the seropositivity observed in **(A)** IIM patients with and without ILD and **(B)** in the SjD cases with and without ILD. Autoantibody levels against 13 of the target proteins are shown where there is seropositivity in at least one subject. Only the results with N-terminal fragment for MDA5 are shown. Color coding denotes relative autoantibody levels in standard deviations above the baseline cut-off value. Autoantibody levels in the patients ranged from low levels (pink) to extremely high autoantibody levels (black). The clear boxes represent seronegative responses with the autoantigens in each subject. Physician global activity score (PGA) is shown for the IIM subjects on the left side of the panel.

To further understand the heterogeneous immunoreactivity seen in the IIM and SjD patients without and with ILD, a heatmap analysis was generated. From the LIPS testing for 21 autoantibodies, 13 target antigens were seropositive (**Figures 4A, B**). A color code was used to denote the relative number of standard deviations in the autoantibody levels in the disease case sera above the corresponding cut-off value for the healthy controls. In the case of IIM (**Figure 4**), the type of myositis and the presence of ILD is listed along with the corresponding physician global score (PGS) of disease activity. All IIM patients without ILD (n=4) showed no seropositivity to any of the autoantigens measured in the study (**Figure 4**). In contrast, 17 of the 21 IIM-ILD sera showed heterogenous seropositivity to at least one autoantigen in the panel. The relative number of autoantibodies or level of seropositivity seen in the IIM patients did not correlate with the overall physician global disease activity. The most informative myositis-specific autoantibodies were anti-Jo-1 autoantibodies, present in three APM and two ADM patients, followed by anti-MDA5 autoantibodies, present in two ADM and two JDM patients (**Figure 4A**). Of note, seropositivity against the five aminoacyl tRNA-synthetases (Jo1, PL7, PL12, Zo and KS) in the IIM-ILD patients did not overlap with the MDA5 seropositive patients. Ro52 seropositive IIM cases were much more likely to harbor anti-MDA-5 and anti-synthetase autoantibodies (Fisher’s exact test P=0.035). Interestingly, the only IIM patient who had high levels U1-70K autoantibodies had the clinical diagnosis of JPM/SLE and had high levels of autoantibodies against both IFN-α and IFN-ω. This patient was seronegative for the eight other anti-synthetase autoantibodies measured in the study. While anti-CENP-A autoantibody (associated with scleroderma), and anti-Ro-60 (associated with SjD) were not informative in the IIM cohort, anti-Ro-52 autoantibody was present in 52% (n=11) of IIM-ILD patients.

Analysis of the autoantibody seropositivity in the SjD patients showed less immunoreactivity to the full panel of autoantigens (**Figure 4B**). All SjD patients, with and without ILD, showed high levels of Ro52 and Ro60 autoantibodies, whereas only five patients were positive for CENP-A autoantibodies. TRIM38 autoantibodies, associated with increased severity of salivary damage in other published cohorts (32, 37), were identified in 30% (n=5) of non-ILD SjD and 20% (n=2) of SjD-ILD patients and were not useful for identifying patients with ILD. Two autoantibodies showed significant seropositivity in the SjD-ILD group only. Three ILD patients had anti-U1-70K autoantibodies and two had anti-KS autoantibodies, while none of the SjD patients without ILD was seropositive for these two autoantibodies. The two SjD-ILD patients with anti-KS autoantibodies were also positive for anti-Ro52 autoantibodies (**Figure 4B**). In the SjD without ILD group, only one patient had low levels of anti-Jo-1 autoantibodies, who had recognized clinical muscle weakness, but had normal serum creatine kinase levels.

### Measuring Jo-1 autoantibodies longitudinally in treated IIM-ILD patients

**Figure 5:**
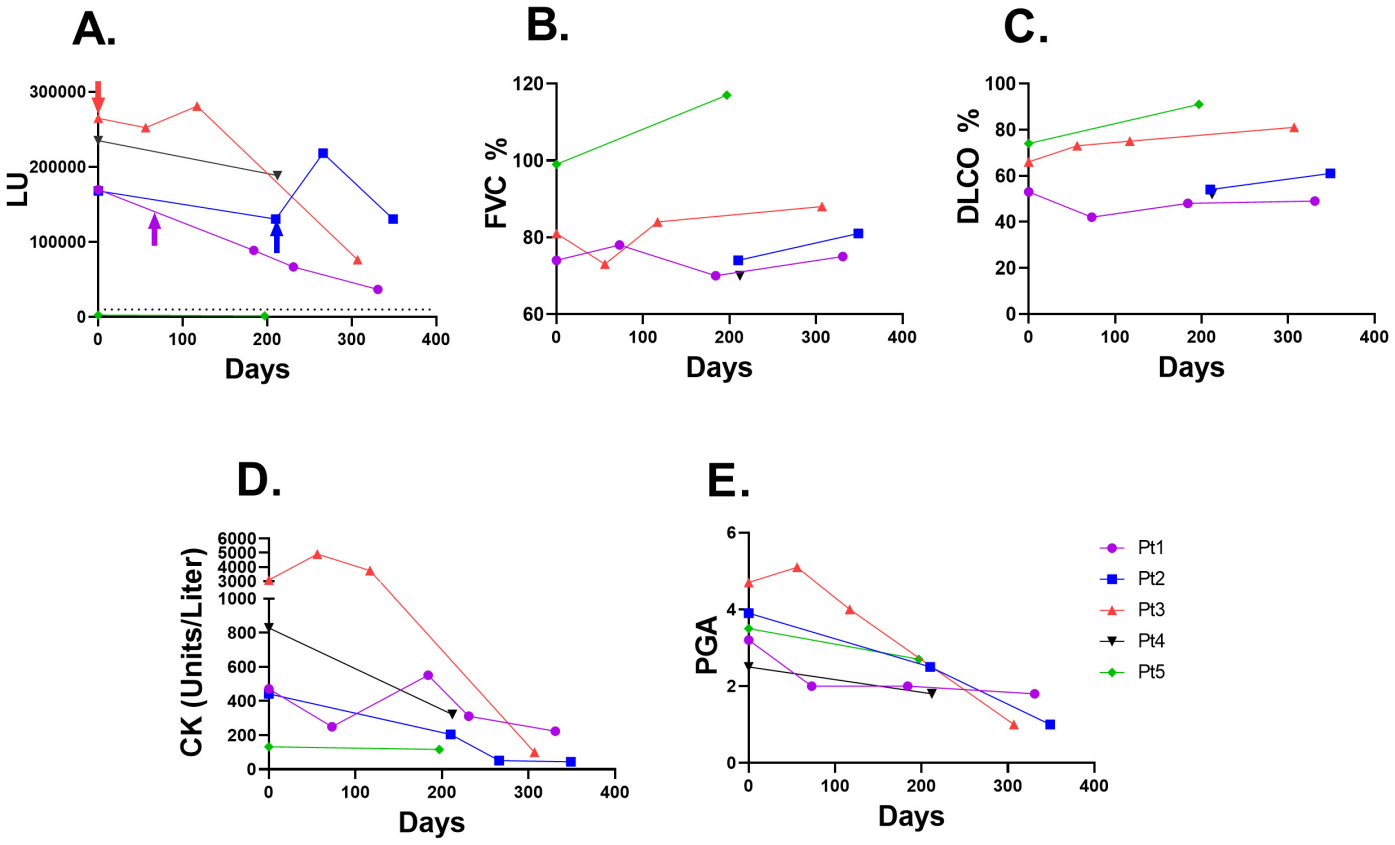
Jo-1 autoantibody trajectory following rituximab immunosuppressive treatment in IIM-ILD patients. **(A)** Jo-1 autoantibody levels (LU) are shown over time in five IIM-ILD patients. Three of the patients (Pt 1-3) received rituximab therapy, in which the time of rituximab administration is shown by the arrow. The cut-off for Jo-1 seropositivity is shown by the dotted line. Patient #5 was seronegative for Jo-1 autoantibodies. **(B)** The corresponding lung function of forced vital capacity (FVC) percent changes **(C)** Diffusion capacity of the lungs for carbon monoxide (DLCO) changes over time **(D)** Creatine kinase (CK) levels and **(E)** Physician global activity (PGA) score for each of the serial samples from the patients shown in panel **(A)**.

Based on prior reports of association of autoantibody levels with disease activity, LIPS was used to evaluate changes in anti-Jo-1 autoantibody levels in IIM patients with sequential samples who had been receiving rituximab immunosuppressive treatment and had clinical and lung function data available. As shown in **Figure 5A**, four of the five subjects were Jo-1 seropositive (pt #5 was Jo-1 seronegative). While the anti-Jo-1 autoantibodies decreased over time, two patients (Pt #1 and Pt #3) showed a marked decrease, with over a 3.5-fold decline in Jo-1 autoantibody levels over the course of one year. The decline in anti-Jo-1 autoantibodies modestly tracked the clinical improvement in pulmonary function testing, in which FVC% (**Figure 5B**) and DLCO% (**Figure 5C**) remained stable or increased in the four anti-Jo1 seropositive patients. Conversely, creatine kinase levels (**Figure 5D**) and physician global activity score (**Figure 5E**) decreased in the patients and tracked with the decline in anti-Jo-1 autoantibody levels.

## Discussion

There is an increasing interest in developing serological biomarkers for screening and monitoring patients with ILD. In this study, we demonstrate the utility of the fluid-phase LIPS assay to detect and quantify a panel of over 14 different autoantibodies, including myositis-specific and myositis-associated autoantibodies in patients with IIM and additional autoantibodies in SjD. The results obtained by LIPS with the IIM patient group for myositis-specific autoantibodies were nearly identical to those obtained via immunoprecipitation by two reference laboratories. First, the detection of anti-MDA-5, -Jo-1, -PL12, and -KS autoantibodies exactly matched the seropositivity status of these other established immunoassays. However, for some of the other targets, there were some discrepancies. While LIPS detected the same sera with PL7 autoantibodies, it also detected an additional seropositive PL7 signal in a Jo-1-positive patient that was not detected by standard immunoprecipitation. While this may be a false positive anti-PL7 autoantibody result, given the rarity of having two anti-synthetase autoantibodies in the same patient, it may also be accurate and reflect the increased sensitivity of the LIPS assay. Moreover, dual anti-tRNA synthetase autoantibody positivity has been reported in a recent study using a multiplex bead array assay (38). LIPS also detected a single Zo seropositive case that was not tested by immunoprecipitation. Beyond providing seropositive/seronegative status, LIPS also provided a wide dynamic range of myositis-specific autoantibody levels that often ranged from 5000 to 3 million LU, a span of three log_10_, in which many of the seropositive autoantibody subjects often had values 100 times higher than the seronegative cases. A recent study identified 16 anti-tRNA synthetase autoantibodies in 72 IIM patients, including the detection of novel autoantibodies that had not been reported in standard assays in patients previously classified as myositis-specific autoantibody negative (38). While the multiplex bead array technology used in that study allowed the identification of novel autoantibodies, it did not quantify autoantibody levels. Nevertheless, these results highlight the possibility that additional anti-synthetase autoantibodies might be included to expand the LIPS assay to measure and quantify autoantibodies in IIM.

Among SjD patients, LIPS demonstrated the presence of U1-70K and KS autoantibodies only in patients with ILD. This is a clinically important finding given that ILD is underdiagnosed in SjD (39). U1-70K autoantibodies, associated with mixed connective tissue, were detected in 3 of the 10 SjD-ILD subjects and none of the SjD subjects without ILD. U1-70K autoantibodies have been found to be a superior marker of mixed connective tissue disease (40) and may be useful in the evaluation of patients with SjD. In our study, LIPS identified and quantified anti-KS autoantibodies in 2 SjD patients, both with ILD. One SjD-ILD subject had quite high levels of KS autoantibodies, and the other subject had lower levels that might be expected to be missed by Western blotting or other immunoassays. While neither of the two KS seropositive SjD-ILD cases reported elevated levels of serum creatine kinase, one individual exhibited muscle weakness. The finding of KS seropositive autoantibodies associated with SjD-ILD is consistent with a recent study by Hosono et al., who screened a cohort of patients with IIM-ILD for KS autoantibodies and detected 9% (16/177 patients) as positive, in which 4 patients had SjD (7). The most striking finding was that half of the anti-KS-positive patients had sicca symptoms, which is one of the key diagnostic criteria for the diagnosis of SS. Four of these patients with sicca symptoms were diagnosed as SjD, which is an uncommon clinical feature in anti-synthetase syndrome. Also, this study found that these patients with KS autoantibodies had less severe myositis and ILD, without muscle weakness and elevated serum creatine kinase levels, and had a relatively favorable prognosis (7). Several case reports have also found KS autoantibodies in SjD. In another study, however, only 1 of 19 patients with anti-KS autoantibodies had SjD (41). The finding that 20% of SjD-ILD had anti-KS autoantibodies in our study may be due to the higher level of sensitivity by LIPS than existing immunoassays. Based on the results, the anti-U1-70K and anti-KS autoantibodies should be further explored as potentially useful markers to be included in the evaluation of patients with SjD, particularly as they might increase the recognition of ILD in this population.

Despite the promising detection and quantification of autoantibodies using LIPS, 30% of the IIM-ILD and 50% of the SjD-ILD subjects did not harbor known autoantibody biomarkers associated with ILD. While there are several factors that could explain this phenomenon (stage of disease, immunosuppressive medications, restricted HLA alleles, among other covariates), it is likely that other autoantibodies that we did not test for, particularly other anti-tRNA-synthetases autoantibodies, are present in these subjects (42). Of note, most of these patients were negative for other autoantibodies associated with autoimmunity and/or lung autoimmunity in other conditions (KCNRG, BPIFB1, TRIM38, CENP-A, interferon-ω, ABLIM, and CDHR) suggesting some specificity for ILD in the context of SjD.

Autoantibodies to TRIM38, a Ro52-related molecule, which have been associated with increased immune infiltrates in the salivary gland and more severe disease in SjD disease (32, 37), were not significantly increased in prevalence in SjD-ILD, highlighting how the immune attack on the lung and salivary gland can occur independently of each other. Interestingly, one JPM/SLE subject who was seronegative for anti-synthetase and anti-MDA5 autoantibodies had anti-U1-70K autoantibodies with high levels of IFN-ω autoantibodies and to a lesser extent with interferon-α autoantibodies. Autoantibodies to interferon-ω and interferon-α are now well-recognized to be associated with high morbidity with lung damage in older male patients infected with SARS-CoV-2 (43) and even contribute to poor outcomes in patients with influenza (44). Interestingly, this patient with anti-interferon autoantibodies had moderate severity of lung disease with dyspnea at rest, cough, pulmonary function tests consistent with restrictive lung disease and the lung biopsy revealed non-specific interstitial pneumonia. Although we did not test the serum of this patient for its capacity to neutralize interferon-ω activity (due to lack of available sample), the relative levels of ant-interferon-ω autoantibodies detected by LIPS are consistent with the possibility that the autoantibodies present in this subject are neutralizing. Future studies screening for a wider range of anti-interferon and other anti-cytokine autoantibodies and their inhibitory effects in a larger cohort of IIM-ILD subjects is warranted.

Previous studies using LIPS have revealed that levels of pathogenic autoantibodies can be used to monitor disease activity during treatment including in patients with membranous nephropathy (45) and Graves’ disease (18). In the present study, we quantified anti-Jo1 autoantibody levels in a small number of subjects with longitudinal data. Our results showed that anti-Jo-1 autoantibody levels decreased over time in patients treated with immunosuppressive therapy whose lung function stabilizes or improves over time. While this has been suggested in a few previous reports in patients with IIM (14, 46–48) and scleroderma (49, 50), the wide dynamic range of levels identified via LIPS may allow us to better assess the role of autoantibody levels as markers of disease activity and treatment response using a highly sensitive, specific, and inexpensive screening tool.

In summary, our study demonstrates LIPS can accurately detect and quantify numerous myositis-specific, myositis-associated, and other lung-related autoantibodies and can quantify a wide range of autoantibody levels in patients with IIM and SjD. Given the known challenges with standard assays measuring non-Jo-1 autoantibodies (16), LIPS is a promising assay that may have a significant role in clinical practice. Furthermore, in SjD, LIPS led to the identification of other relevant non-SjD-related autoantibodies which reflect important clinical features of disease activity for ILD. This will facilitate the characterization of clinically relevant subsets of patients. Limitations of our study include the small sample sizes of each patient group and the lack of concomitant measures of lung function or symptoms on the day of blood sampling. It is also possible that we underestimated the seroprevalence of a few of the anti-synthetase autoantibodies due to the use of protein fragments instead of full-length protein, but to evaluate will require testing a larger cohort of samples. Future studies applying LIPS to evaluate a wider range of autoantibodies in a large cohort of deeply phenotyped ILD subjects over time are warranted to assess whether quantitative autoantibody levels can be useful biomarkers in the stratification, disease monitoring, and treatment responses in patients with autoimmune ILD.

## Data Availability

The raw data supporting the conclusions of this article will be made available by the authors, without undue reservation.
